# Best diagnostic accuracy of sepsis combining SIRS criteria or qSOFA score with Procalcitonin and Mid-Regional pro-Adrenomedullin outside ICU

**DOI:** 10.1038/s41598-020-73676-y

**Published:** 2020-10-06

**Authors:** Silvia Spoto, Edoardo Nobile, Emanuele Paolo Rafano Carnà, Marta Fogolari, Damiano Caputo, Lucia De Florio, Emanuele Valeriani, Domenico Benvenuto, Sebastiano Costantino, Massimo Ciccozzi, Silvia Angeletti

**Affiliations:** 1 Diagnostic and Therapeutic Medicine Department, University Campus Bio-Medico, Via Alvaro del Portillo 200, 00128 Rome, Italy; 2 Unit of Clinical Laboratory Science, University Campus Bio-Medico, Rome, Italy; 3 Department of Surgery, University Campus Bio-Medico, Rome, Italy; 4 Unit of Medical Statistics and Molecular Epidemiology, University Campus Bio-Medico, Rome, Italy

**Keywords:** Biomarkers, Health care, Medical research

## Abstract

Early diagnosis and treatment significantly reduce sepsis mortality. Currently, no gold standard has been yet established to diagnose sepsis outside the ICU. The aim of the study was to evaluate the diagnostic accuracy of sepsis defined by SIRS Criteria of 1991, Second Consensus Conference Criteria of 2001, modified Second Consensus Conference Criteria of 2001 (obtaining SIRS Criteria and SOFA score), Third Consensus Conference of 2016, in addition to the dosage of Procalcitonin (PCT) and MR-pro-Adrenomedullin (MR-proADM). In this prospective study, 209 consecutive patients with clinical diagnosis of sepsis were enrolled (May 2014–June 2018) outside intensive care unit (ICU) setting. A diagnostic protocol could include SIRS criteria or qSOFA score evaluation, rapid testing of PCT and MR-proADM, and SOFA score calculation for organ failure definition. Using this approach outside the ICU, a rapid diagnostic and prognostic evaluation could be achieved, also in the case of negative SIRS, qSOFA or SOFA scores with high post-test probability to reduce mortality and improve outcomes.

## Introduction

Sepsis is the first cause of death for infection accounting for 17% of intra-hospital mortality and reaching 26% in case of septic shock^1^ with estimated costs for over 24 billion of dollar per year^2^. In 2016, the Third international Consensus Conference (Sepsis-3) defined sepsis as a “life-threatening organ dysfunction caused by a dysregulated host response to infection” removing among diagnostic criteria the presence of the systemic inflammatory response syndrome (SIRS), previously used in the Sepsis-1 and Sepsis-2 Consensus^3–6^.

Septic shock was identified by the presence of at least one of persistent hypotension requiring vasopressor administration to maintain MAP ≥ 70 mmHg and serum lactate > 2 mmol/L (> 18 mg/dL) despite adequate blood volume expansion^6^. The Third Consensus Conference (Sepsis-3) established that, in presence of suspected or documented infection, an increase of Sequential Sepsis-related Organ Failure Assessment (SOFA) score in intensive care unit (ICU) ≥ 2 from baseline have to be considered diagnostic for sepsis^6^. An increase of the quick SOFA (qSOFA) score ≥ 2 from baseline may be suggestive of sepsis, mainly outside ICU^6^.

SOFA score ≥ 2 was associated with an intra-hospital mortality > 10% with values as high as 40% in case of septic shock^6,7^.

In the Third definition, SOFA and qSOFA replaced SIRS criteria of the 1991 definition (Sepsis-1)^3^ considered too much sensitive and not specific, causing overdiagnosis and inappropriate use of antibiotics. Patients admitted to the Emergency Department could present SIRS in many situations including metabolic and endocrine diseases, cancer, respiratory syndromes, infections, trauma and ischemia^8^. Factors as drugs or disease altering body temperature, cardiac or respiratory frequencies and leukocytes count can determine SIRS. Furthermore, the identification of the microbiological cause of sepsis is achieved in less than 50% of patients^9^ and only 30% of bacteremia are microbiologically documented^10^.

Since 2001, the Second Consensus Conference of Sepsis recommended adding the use of biomarkers to SIRS criteria to overcome these limits^4,5,11^. During infection, indeed, PCT has the ability to discriminate between infectious and not-infectious SIRS, and to guide antimicrobial therapy and follow-up^12–15^. However, PCT increase during infection by Gram-positive or fungal pathogens or antimicrobial treatment could be limited^16–20^.

Mid-regional pro-adrenomedullin (MR-proADM) has been recently proposed for sepsis diagnosis and prognosis, also providing etiological information^12,21–24^. Its levels significantly relate with septic patients’ outcomes showing good relation with prognosis and mortality rate^25^. Despite the availability of PCT and MR-proADM may be hampered mainly in the low income countries, they presented lower turnaround time and lower costs than other biomarkers of sepsis (interleukins, cytokines, and others biomarkers) that had similar diagnostic capability.

In sepsis and septic shock, MR-proADM compared to other well-known biomarkers or clinical scores, showed a prognostic accuracy higher than those of PCT, IL-6, CRP or clinical scores as Acute Physiology and Chronic Health (APACHE)^23,24,26^. Christ-Crain et al. analyzed MR-proADM levels in septic patients admitted to intensive care unit (ICU) showing a significantly higher correlation with sepsis severity than PCT and CRP^26^. Recently, Kim et al., reported a significant correlation between MR-proADM levels and septic shock, need for vasopressor, and 30-day mortality, suggesting its inclusion in the panel of biomarkers that may be useful for diagnosis and treatment management of critical patients in ICU^27^.

The combination of MR-proADM with other biomarkers, especially PCT, was proposed in previous studies^21–23,28^.

The combined measurement of PCT and MR-proADM significantly improved sepsis diagnosis, mainly in case of Gram-positive or fungal sepsis where PCT alone could present a lower positive predictive value (PPV)^12^.

Actually, the most significant approach to reduce sepsis-related mortality is based on early diagnosis by adequate microbiological cultures collection and administration of empirical antibiotic treatment within 3 h from clinical suspicion^29^. To reduce antimicrobial resistance, however, would be desirable to administer an appropriate antimicrobial treatment basing on the results of microbiological specimen even if these latter yield positive results just in less than 50% of cases^9,10^. Two recent systematic review and meta-analysis highlighted as an earlier than delayed antimicrobial treatment administration seemed not to reduce mortality in patients with septic shock, despite the effect of other specific treatment were not considered within the analysis^30,31^. These contrasting results is responsible for the lack of standardized treatment strategies.

The aim of the study was to evaluate the diagnostic accuracy of sepsis defined by SIRS Criteria of 1991, Second Consensus Conference Criteria of 2001, modified Second Consensus Conference Criteria of 2001 (obtaining SIRS Criteria and SOFA score), Third Consensus Conference of 2016, in addition to the dosage of Procalcitonin (PCT) and MR-pro-Adrenomedullin (MR-proADM).

## Methods

### Design and setting

This study was approved by the Ethical Committee of the University Hospital Campus Bio-Medico of Rome. All methods were performed in accordance with the relevant guidelines and regulations. Informed consent was obtained from all patients prior enrollment in the study.

### Patients selection and study design

**Figure 1:**
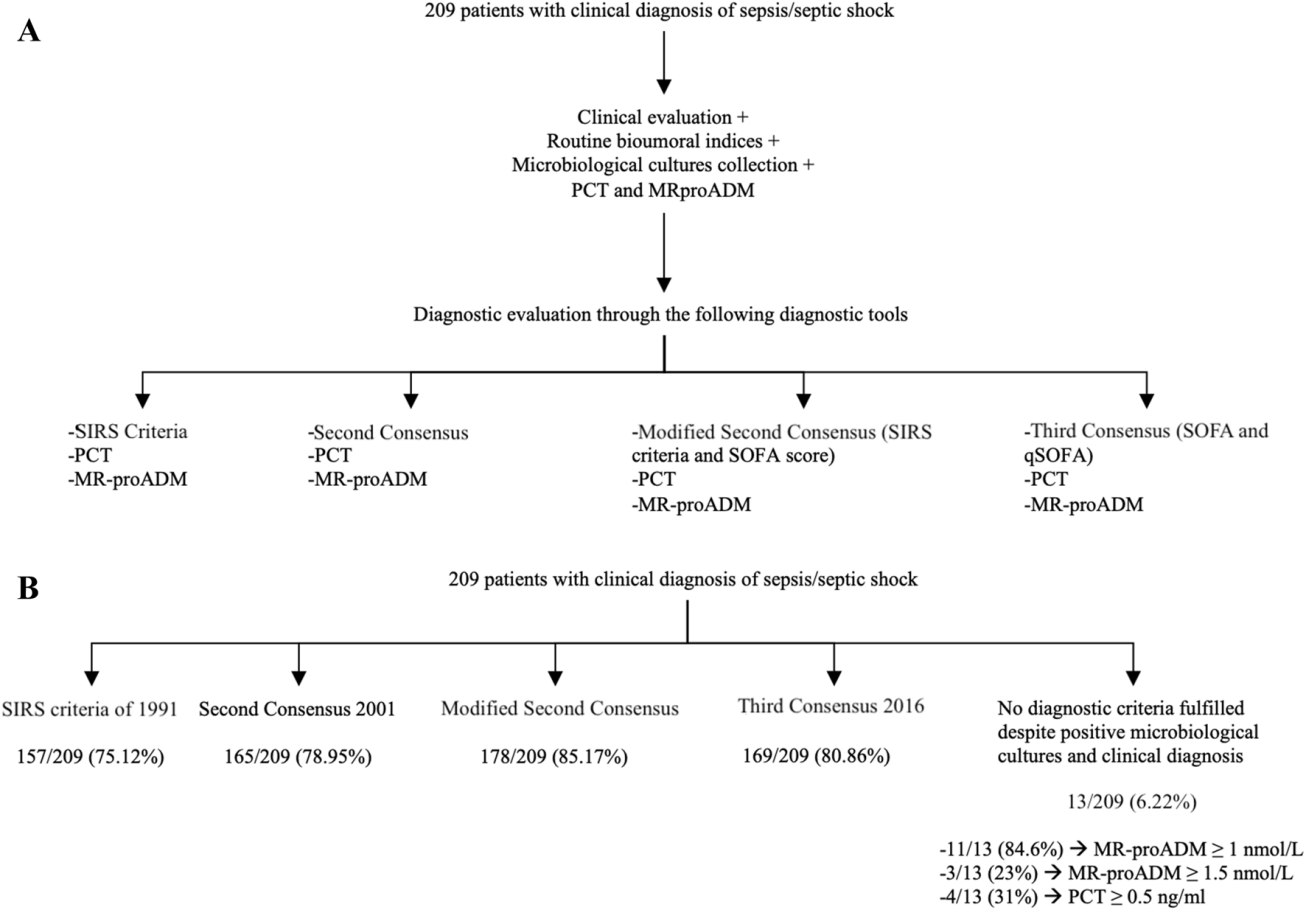
Algorithm used for sepsis clinical diagnosis (**A**) and diagnostic evaluation (**B**).

This prospective study was performed on consecutive enrolled patients with clinically suspected sepsis or septic shock admitted to the Diagnostic and Therapeutic Medicine Department and General Surgery of the University Hospital Campus Bio-Medico of Rome, between May 2014 and June 2018. Data were retrospectively evaluated comparing the diagnostic accuracy of sepsis as defined by SIRS criteria of 1991, Second Consensus Conference Criteria of 2001, modified Second Consensus Conference Criteria of 2001, Third Consensus Conference of 2016, in addition to the dosage of PCT and MR-proADM (Fig. 1A). Modified Second Consensus Conference Criteria of 2001 were obtained using SIRS Criteria of 1991 plus SOFA score of 2016^3–6^.

Inclusion criteria comprehended the clinical suspicion of sepsis and septic shock. Exclusion criteria were the lack of informed consent and pregnancy. At inclusion (Day 0), demographic characteristics such as age, gender, prior or current use of antibiotics, immunosuppressive treatments, immune status (active malignancy or other causes of an immunocompromised state), comorbidities and clinical presentation were recorded. For each patients a physical examination including cardiac, abdominal, respiratory and neurological evaluations was performed.

The real-world control group included fifty patients admitted to the Diagnostic and Therapeutic Medicine Department of Campus Bio-Medico of Rome for cardiac, kidney, liver, pulmonary and cancer diseases being responsible for a non-infectious related SIRS, qSOFA, or SOFA criteria positivity.

### Clinical and laboratory parameters, blood gas analysis, blood and microbiological cultures

The following clinical and laboratory parameters have been collected: body temperature, blood pressure, heart and respiratory rate, complete blood counts (CBC), PCT, MR-proADM, bilirubin, creatinine, lactate, PaO_2_/FIO_2_, and blood and microbiological cultures at the diagnosis and when clinically necessary.

#### PCT and MR-proADM plasma measurement

PCT and MR-proADM plasma concentrations were measured by an automated Kryptor analyzer, using a time-resolved amplified cryptate emission (TRACE) technology assay (Kryptor PCT; Brahms AG; Hennigsdorf, Germany), with commercially available immunoluminometric assays (Brahms)^21–24^.

#### Blood and microbiological cultures

Blood specimens from patients were collected in BACTEC bottles containing anaerobic or aerobic broth and resins. Blood culture bottles (BC) were incubated in BACTEC FX instrument (Becton Dickinson, Meylan, France) until they resulted positive for bacterial growth or for a maximum of 5 days. Positive BC samples were cultivated in selective agar media. Growing colonies were identified by MALDI-TOF^22^. Selective and non selective media were used for microbiological cultures.

### Sepsis diagnosis

Patients with suspected sepsis or septic shock included in the study were retrospectively evaluated by SIRS criteria of 1991, Second Consensus Conference Criteria of 2001, the modified Second Consensus Conference Criteria of 2001 (obtaining SIRS Criteria and SOFA score), Third Consensus Conference Criteria of 2016, in addition to the dosage of PCT and MR-proADM^3–6^. A SOFA or a qSOFA scores ≥ 2 from baseline has been considered diagnostic of sepsis.

### Statistical analysis

Data were analysed using Med-Calc 11.6.1.0 statistical package (MedCalc Software, Mariakerke, Belgium). Plasma levels of PCT, MR-proADM, SOFA and qSOFA score values, Second Consensus Conference Criteria, modified Second Consensus Conference Criteria and SIRS criteria in septic patients and real-life control patients were compared using the non-parametric Mann–Whitney’s test; *p* value < 0.05 were considered as significant. Receiver operating characteristic (ROC) analysis was performed among independent variables associated with sepsis to define the cutoff point for plasma PCT, MRproADM, lactate, SOFA and qSOFA score values, SIRS criteria of 1991, Second Consensus Conference Criteria, modified Second Consensus Conference Criteria and to define their diagnostic accuracy for sepsis prediction. ROC curves and areas under the curve (AUCs) were calculated for all markers and compared in patients with sepsis or septic shock versus real-life control patients^32^.

χ^2^ for proportions test was used to compare the relative percentage of patients with positivity and/or negativity to SIRS criteria, SOFA score, qSOFA score, PCT and MR-proADM. *p* value < 0.05 were considered as significant.

Pretest odds, posttest odds, and the consequent posttest probability and χ^2^ test for proportions have been computed to investigate whether combination of PCT, MR-proADM, lactate, SOFA, qSOFA scores, SIRS criteria of 1991, Second Consensus Conference Criteria, modified Second Consensus Conference Criteria improves post-test probability^33^.

## Results

### Patients characteristics

The demographic and clinical characteristics of the study group including 209 patients with sepsis and of the 50 real-world control group patients are reported in Table 1. The control group included patients with cardiac, kidney, liver, pulmonary and cancer diseases being responsible for a non-infectious related SIRS, qSOFA, or SOFA criteria positivity.

**Table 1:** Demographic and clinical characteristics of study and control groups.

Variables	Study group = 209	Control group = 50	*p* value*
Median age (IQR)	***72 (64–80)***	***74 (66–82)***	***0.06***
Sex male n (%)	*107 (51)*	*25 (50)*	*0.89*
Hypertension n (%)	105 (50)	26 (51)	*0.89*
Hyperlipidemia n (%)	42 (20)	12 (23.5)	*0.59*
Diabetes mellitus n (%)	44 (21)	10 (19.6)	*0.82*
Hypertensive cardiopathy n (%)	28 (13.4)	10 (19.6)	*0.26*
Ischemic cardiopathy n (%)	26 (12.4)	5 (9.8)	*0.61*
Degenerative cardiopathy n (%)	15 (7.2)	4 (7.8)	*0.88*
Chronic cardiac failure n (%)	9 (4.3)	10 (19.6)	***0.0002***
Acute kidney injury (AKI) n (%)	28 (13.4)	0 (0)	***0.0062***
Chronic kidney disease (CKD) n (%)	34 (16)	8 (15.7)	*0.95*
Chronic obstructive pulmonary disease (COPD) n (%)	31 (14.8)	9 (17.6)	*0.62*
Viral hepatitis n (%)	13 (6)	0 (0)	*0.08*
Cirrhosis n (%)	7 (1.9)	3 (5.8)	*0.12*
Solid neoplasia n (%)	59 (28)	15 (3)	***0.0002***
Hematologic neoplasia n (%)	7 (3)	3 (6)	*0.30*
Autoimmune disease/immunosuppressive therapy n (%)	17 (8)	7 (14)	*0.18*
Antimicrobial therapy on course	162 (77.5)	n.a	–
Septic shock	84 (40)	n.a	–
30-days mortality	38 (18)	n.a	–
30-days mortality for sepsis	10 (8)	n.a	–
30-days mortality for septic shock	28 (33)	n.a	–
90-days mortality	48 (23)	n.a	–
90-days mortality for sepsis	16 (13)	n.a	–
90-days mortality for septic shock	32 (38)	n.a	–

Bold italics identify statistically significant *p*-values.

*χ^2^ for proportion: *p* value < 0.05 were considered statistically significant; *n.a*.  not available.

Septic patients and control group were similar except for the presence of chronic cardiac failure that was significantly more represented in control population (*p* = 0.0002), acute kidney injury^34^ and solid cancer that were more prevalent in study group (*p* = 0.0062 and *p* = 0.0002, respectively). Septic shock was diagnosed in 82 out of 209 (39%) patients (Table 1). In 162/209 (77.5%) patients antimicrobial therapy was administered before sepsis diagnosis (Table 1). 30-day mortality was 8% in patients with sepsis, reaching values as high as 33% in case of septic shock, whereas 90-days mortality was 13% in sepsis and 38% in septic shock (Table 1).

### Sepsis diagnosis

Sepsis was diagnosed in 157/209 (75.12%) patients by SIRS criteria of 1991, in 165/209 (78.95%) by Second Consensus Conference Criteria of 2001, in 178/209 (85.17%) by modified Second Consensus Conference Criteria of 2001, and in 169/209 (80.86%) by Third Consensus Conference Criteria of 2016 (Fig. 1B).

In 13/209 (6.22%) patients no criteria were fulfilled for sepsis diagnosis despite a blood culture positive for microbiological isolates. In particular, 3/13 (23%) were positive for bacterial endocarditis by *Streptococcus sanguinis*, *Kytococcus schroeteri* and *Staphylococcus epidermidis*; 5/13 (31%) presented urosepsis by *Enterococcus faecium*, *Klebsiella pneumoniae*, *Staphylococcus aureus* and *Candida albicans*; 1/13 (7.70%) had diagnosis of pneumonia by *Enterococcus faecalis*; 2/13 (15.40%) had diagnosis of osteomyelitis by *Staphylococcus aureus* and *Staphylococcus hominis*; 2/13 (15.4%) had diagnosis of catheter related bloodstream infection (CRBSI) by *Raoultella ornithinolytica*, *Providencia stuartii*, *Proteus mirabilis* and *Klebsiella oxytoca*. In these patients, 11/13 (84.62%) had positive bacteremia with MR-proADM values above the cut-off of ≥ 1 nmol/L and 3/13 (23%) above the values of > 1.5 nmol/L. PCT values was ≥ 0.5 ng/mL in 4/13 (31%) patients.

In 188/209 (90%) patients SIRS criteria, qSOFA, SOFA scores, or all of them were < 2. Particularly, 41/127 (32.33%) patients with sepsis had < 2 SIRS criteria, 32/125 (25.62%) had a SOFA score < 2, 35/125 (28%) had SIRS criteria and qSOFA score < 2, 30/125 (24%) had SOFA and qSOFA scores < 2, and 16/125 (12.81%) had SIRS criteria, qSOFA and SOFA scores < 2. Among patients with septic shock, 11/82 (13.43%) patients with septic shock had SIRS criteria < 2 and 10/82 (12.25%) SIRS criteria and qSOFA score < 2 (Table 2).

**Table 2:** Percentage of septic (S) and septic shock (SS) patients negative for SIRS criteria (SIRS < 2) or negative for SIRS criteria (SIRS < 2) plus qSOFA score (qSOFA < 2) with positivity to other markers.

S patients	SOFA ≥ 2	qSOFA ≥ 2	PCT ≥ 0.5 ng/mL	MR-proADM ≥ 1 nmol/L
SIRS < 2 (n = 41)	22/41 (54%)	4/41 (10%)	21/41 (51%)	32/41 (78%)
SIRS < 2 + qSOFA < 2 (n = 35)	19/35 (54%)	–	20/35 (57%)	35,739 (89%)
SOFA < 2 (n = 32)	–	1/32 (3%)	9/32 (28%)	17/32 (53%)
SOFA < 2 + qSOFA < 2 (n = 30)	–	–	14/30 (%)	28/30 (%)
SIRS < 2 + SOFA < 2 + qSOFA < 2 (n = 16)	–	–	7/16 (%)	13/16 (%)

SS patients	SOFA > 2	qSOFA > 2	PCT ≥ 0.5 ng/mL	MR-proADM ≥ 1 nmol/L
SIRS < 2 (n = 11)	11/11 (100%)	3/11 (27%)	7/11 (64%)	11/11 (100%)
SIRS < 2 + qSOFA < 2 (n = 10)	8/10 (80%)	–	6/10 (60%)	10/10 (100%)

### PCT, MR-proADM, SIRS criteria, qSOFA and SOFA score values in study population

Median values, interquartile ranges (25th percentile and 75th percentile), and Mann–Whitney’s comparison of the different variables are reported in Table 3. In particular, the median number of SIRS criteria registered was two, the median qSOFA score was 1, the median SOFA score was 4, PCT and MR-proADM median levels were 1.16 ng/mL and 2.55 nmol/L, respectively. All variables resulted significantly higher in septic patients than control group (*p* < 0.0001) (Table 3).

**Table 3:** Median values, interquartile ranges (25th percentile and 75th percentile), and Mann–Whitney’s comparison of the different variables registered in the study and control groups.

Median values (IQR)	Septic patients = 209	Control group = 50	*p* value*
SIRS criteria	2 (2–3)	1 (0–1)	< 0.0001
SOFA score	4 (2–6)	1 (0–2)	< 0.0001
qSOFA score	1 (0–2)	0 (0–0)	< 0.0001
PCT ng/mL	1.16 (0.31–5.10)	0.06 (0.05–0.15)	< 0.0001
MR-proADM nmol/L	2.55 (1.72–4.38)	1.14 (0.8–1.51)	< 0.0001

*χ^2^ Mann–Whitney’s comparison. *p* value < 0.05 were considered statistically significant.

### ROC curves analysis and areas under the curves (AUCs)

In septic patients, the AUCs values for SIRS criteria, Second Consensus Conference Criteria, modified Second Consensus Conference Criteria, qSOFA and SOFA score are reported in Table 4.

**Table 4:** ROC Curves analysis: Areas under the Curves (AUCs) values for SIRS criteria, Second Consensus Conference Criteria, modified Second Consensus Conference Criteria, SOFA score and qSOFA score values in the study population.

Variables	AUC value	Cut-off	Sens%	Spec%	LR +	*p* value
SIRS criteria	0.85	> 2	75.12	84.31	4.79	< 0.0001
Second Consensus Conference Criteria	0.86	> 4				< 0.0001
Modified Second Consensus Conference Criteria	0.85	> 4				< 0.0001
SOFA score	0.82	> 2	66.51	82.35	3.77	< 0.0001
qSOFA score	0.77	> 2				< 0.0001
PCT ng/mL	0.93	> 0.5	67.94	98.04	34.65	< 0.0001
MR-proADM nmol/L	0.85	> 1.5	83.0	76.47	3.53	< 0.0001

**Figure 2:**
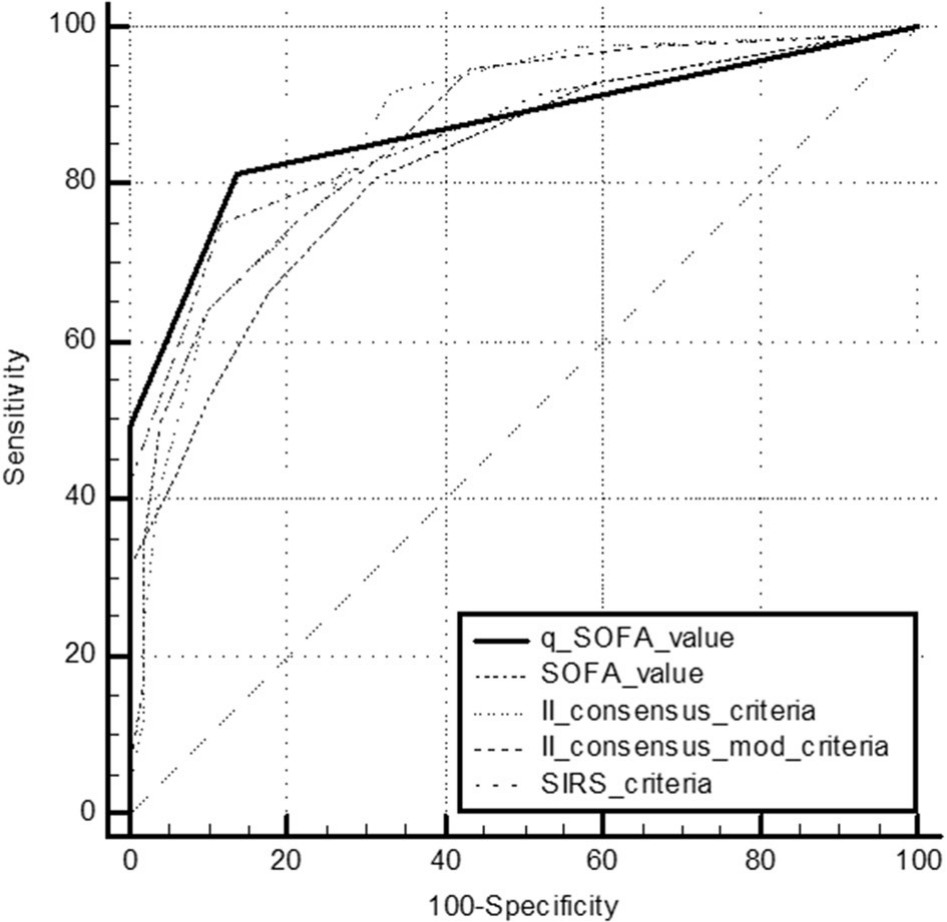
ROC curves comparison between SIRS, Second Consensus Conference, modified Second Consensus Conference Criteria, SOFA and qSOFA score values.

**Figure 3:**
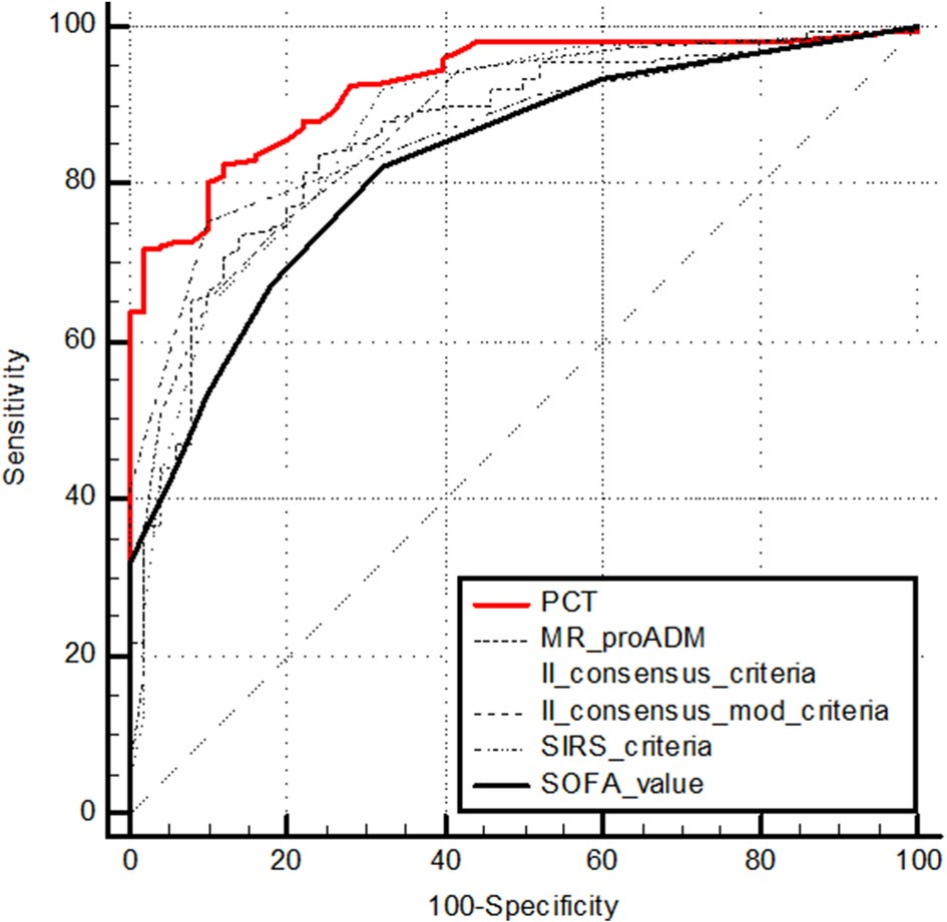
ROC curves comparison between PCT, MR-proADM, SIRS, Second Consensus Conference, modified Second Consensus Conference Criteria, and SOFA score values.

ROC curves comparison between SIRS criteria, Second Consensus Conference Criteria, modified Second Consensus Conference Criteria, qSOFA and SOFA score has been reported in Fig. 2. Any statistically significant difference has been highlighted. Adding PCT and MR-proADM to the ROC curve analysis, PCT AUC was significantly higher (*p* < 0.05) than all other variables (Fig. 3; Table 4).

**Figure 4:**
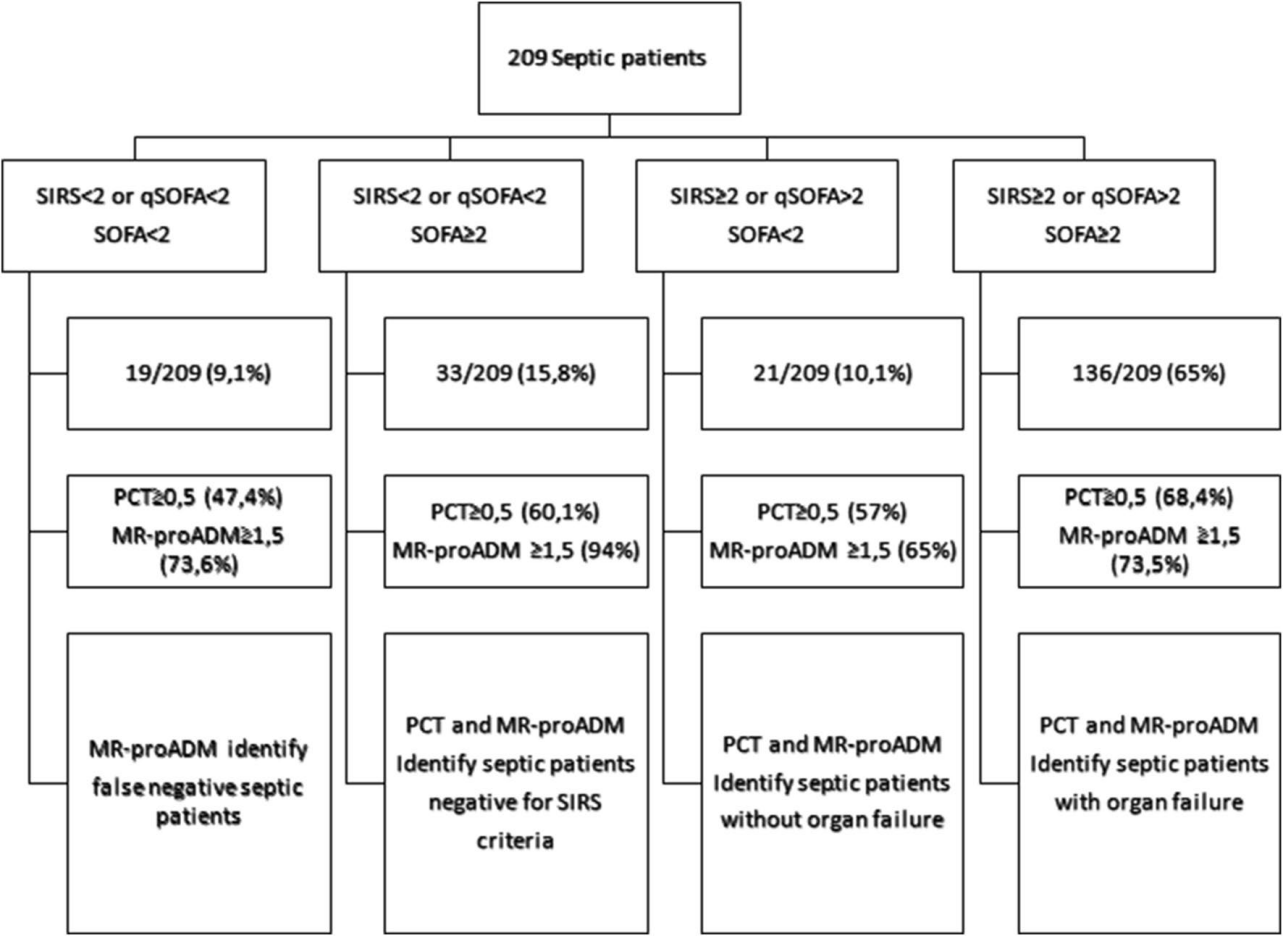
PCT and MR-proADM biomarkers evaluation in septic patients stratified by SIRS, qSOFA and SOFA score.

Based on SIRS criteria of 1991, Second Consensus Conference Criteria, modified Second Consensus Conference Criteria, qSOFA and SOFA score ROC curve overlapping, septic patients were stratified using SIRS criteria of 1991 or qSOFA score, easy and rapid to calculate, and SOFA score, the actual diagnostic tool. PCT and MR-proADM biomarkers evaluation in septic patients stratified by SIRS, qSOFA and SOFA score were reported in Fig. 4. In particular, 19/209 (9.11%) patients presented SIRS criteria < 2, qSOFA score < 2 and SOFA score < 2. Among these patients, 9/19 (47.47%) had PCT levels ≥ 0.5 ng/mL, 14/19 (73.63%) MR-proADM > 1.5 nmol/L and 15/19 (78.92%) positive blood culture with documented microbiological isolates. In 33/209 (15.78%) patients the fulfilled SIRS criteria was < 2, qSOFA score < 2 but SOFA score value ≥ 2; within this group, 20/33 (60%) of patients showed PCT ≥ 0.5 ng/mL and 31/33 (94%) MR-proADM > 1.5 nmol/L. In 21/209 (10%) patients SIRS criteria were ≥ 2, qSOFA score ≥ 2 but SOFA score values < 2. Among these, 12/21 (57%) had PCT levels ≥ 0.5 ng/mL and 14/21 (65%) MR-proADM > 1.5 nmol/L. 136/209 (65%) patients had SIRS criteria ≥ 2, qSOFA score ≥ 2 and SOFA score ≥ 2. In these patients, 93/136 (68.46%) had PCT levels ≥ 0.5 ng/mL and 100/136 (73.56%) MR-proADM > 1.5 nmol/L.

Globally, in 45/209 (21.56%) septic patients, SIRS criteria and qSOFA score were < 2. In these patients some confounding factors potentially influencing SIRS criteria and qSOFA evaluation were present. In particular, 41/45 (92%) of patients were receiving drugs with negative chronotropic effect such as beta-blockers, calcium antagonists or other antiarrhythmic drugs with impact on cardiac rate. In 6/45 (13.51%) a pacemaker DDD for bradyarrhythmia was present, affecting cardiac rate. In 36/45 (81%) antimicrobial therapy and in 4/45 (8.01%) paracetamol were administered affecting both body temperature or heart rate increase. Regarding influence on respiratory rate, 6/45 (13.52%) were receiving benzodiazepine treatment and 3/45 (6.70%) chronic oxygen therapy.

### χ^2^ test for proportions in patients with sepsis and septic shock in case of negativity for SIRS criteria: SOFA score, qSOFA score, PCT and MR-proADM comparison

Forty-one patients with sepsis and 11 patients with septic shock presented SIRS criteria < 2. In Table 5, the percentage of patients with sepsis and septic shock with SIRS criteria < 2 has been stratified by SOFA score, qSOFA score, PCT and MR-proADM. In case of sepsis, 56% of patients presented SOFA score ≥ 2, 10% qSOFA ≥ 2, 54% PCT ≥ 0.5 ng/mL, and 82% MR-proADM ≥ 1 nmol/L. In septic shock, 100% of patients presented SOFA score ≥ 2, 27% qSOFA ≥ 2, 64% PCT ≥ 0.5 ng/mL, and 100% MR-proADM ≥ 1 nmol/L (Table 2).

**Table 5:** χ^2^ for proportions in patients with sepsis (S) and septic shock (SS) and negative for SIRS criteria (SIRS < 2): SOFA, qSOFA, PCT and MR-proADM comparison.

Patients with sepsis and SIRS < 2 (n = 41)	SOFA ≥ 2 (56%)	qSOFA ≥ 2 (10%)	PCT ≥ 0.5 ng/mL (54%)	MR-proADM ≥ 1 nmol/L (82%)
MR-proADM ≥ 1 nmol/L (82%)	***p* = 0.0014**	***p* < 0.0001**	***p* = 0.0084**	–
PCT ≥ 0.5 ng/mL (54%)	*p* = 0.96	***p* < 0.0001**	–	***p* = 0.0084**
SOFA ≥ 2 (56%)	–	***p* < 0.0001**	*p* = 0.91	***p* = 0.0014**
qSOFA ≥ 2 (10%)	***p* < 0.0001**	–	***p* < 0.0001**	***p* < 0.0001**

Patients with septic shock and SIRS < 2 (n = 11)	SOFA ≥ 2 (100%)	qSOFA ≥ 2 (27%)	PCT ≥ 0.5 ng/mL (64%)	MR-proADM ≥ 1 nmol/L (100%)
MR-proADM ≥ 1 nmol/L (100%)	*p* = 0.15	***p* = 0.001**	***p* = 0.006**	–
PCT ≥ 0.5 ng/mL (54%)	*p* = 0.09	*p* = 0.11	–	***p* = 0.006**
SOFA ≥ 2 (85%)	–	***p* = 0.002**	*p* = 0.09	*p* = 0.15
qSOFA ≥ 2 (23%)	***p* = 0.002**	–	*p* = 0.11	***p* = 0.001**

Bold identify statistically significant *p*-values.

χ^2^ test for proportions analysis showed that in septic patients with SIRS criteria < 2, MR-proADM is significantly superior to SOFA score (*p* = 0.0014), qSOFA score (*p* < 0.0001) and PCT (*p* = 0.0084) (Table 5). SOFA score and PCT are both significantly superior to qSOFA score (*p* < 0.0001) (Table 5). In septic shock patients, MR-proADM is significantly superior to PCT (*p* = 0.006) and qSOFA score (*p* = 0.0001) but it is comparable to SOFA score (*p* = 0.15) (Table 5). SOFA score is significantly superior to qSOFA score (*p* = 0.002) (Table 5).

### χ^2^ test for proportions in patients with sepsis and septic shock in case of negativity for SIRS criteria plus qSOFA score: SOFA, PCT and MR-proADM comparison

In 35 patients with sepsis and 10 patients with septic shock SIRS criteria and qSOFA score were < 2. Stratifying septic patients for SOFA score, PCT and MR-proADM values (Table 2), 54% presented SOFA score ≥ 2, 57% PCT ≥ 0.5 ng/mL, and 89% MR-proADM ≥ 1 nmol/L. In case of septic shock, SOFA score was ≥ 2 in 80%, PCT was ≥ 0.5 ng/mL in 60% and MR-proADM was ≥ 1 nmol/L in 100% (Table 2).

χ^2^ test for proportions analysis showed that in septic patients with SIRS criteria and qSOFA score < 2, MR-pro ADM was superior to SOFA score (*p* = 0.001) and PCT (*p* = 0.002) (Table 6). In septic shock, MR-proADM was comparable to SOFA score (*p* = 0.34) but superior to PCT (*p* = 0.03) (Table 6).

**Table 6:** χ^2^ for proportions in patients with sepsis and septic shock and negative at SIRS criteria (SIRS < 2) and qSOFA < 2: SOFA, PCT and MR-proADM comparison.

Patients with sepsis SIRS < 2 + qSOFA < 2 (n = 35)	SOFA ≥ 2 (54%)	PCT ≥ 0.5 ng/mL (57%)	MR-proADM ≥ 1 nmol/L (89%)
SOFA ≥ 2 (54%)	–	*p* = 0.80	***p* = 0.001**
PCT ≥ 0.5 ng/mL (57%)	*p* = 0.80	–	***p* = 0.002**
MR-proADM ≥ 1 nmol/L (89%)	***p* = 0.001**	***p* = 0.002**	–

Patients with septic shock SIRS < 2 + qSOFA < 2 (n = 10)	SOFA > 2 (80%)	PCT ≥ 0.5 ng/mL(60%)	MR-proADM ≥ 1 nmol/L (100%)
SOFA ≥ 2 (80%)	–	*p* = 0.34	*p* = 0.14
PCT ≥ 0.5 ng/mL (60%)	*p* = 0.40	–	***p* = 0.03**
MR-proADM ≥ 1 nmol/L (100%)	*p* = 0.34	***p* = 0.03**	–

Bold identify statistically significant *p*-values.

### Combined PCT, MR-proADM, SIRS criteria, qSOFA and SOFA scores measurement in sepsis diagnosis: the post-test probability

Post-test probability analysis was performed to define the diagnostic value derived from the use of the single clinical score or criteria or of the single biomarker as well as from the combination of all the clinical parameters and laboratory markers. The results of the post-test probability are reported in Table 7. The association between PCT measurement and SIRS criteria or PCT and qSOFA score reached a diagnostic accuracy of 99.9%.

**Table 7:** Post-test probability analysis used to define the diagnostic value derived from the combined use of PCT, MR-proADM, SOFA score and SIRS criteria in patients with sepsis or septic shock.

Diagnostic test	LR +	Post-test probability
PCT	34.65	0.990
SIRS criteria	9.79	0.970
qSOFA score	5.93	0.960
MR-proADM	3.48	0.940
SOFA score	3.77	0.940

Test combination	Post-test probability	
SIRS criteria + PCT	0.999	
qSOFA + PCT	0.999	
PCT + MR-proADM	0.997	
SOFA score + PCT	0.997	
SOFA score + PCT + MR-proADM	0.999	
SIRS criteria + PCT + MR-proADM	0.999	
qSOFA score + PCT + MR-proADM	0.999	

The combination of PCT, SIRS or qSOFA and MR-proADM provide a diagnostic and prognostic evaluation in 99.9% of patients with a turnaround time of about 45 min, whereas the combination of PCT, SOFA score and MR-proADM reaching comparable accuracy (99.9%) requires a turnaround time of about 90 min.

## Discussion

The physiopathology of sepsis highlights the need of unambiguous diagnostic criteria for a rapid patients identification and adequate therapy administration, within one hour from symptoms presentation^35^. Sepsis definition and diagnostic criteria proposed from 1991 until now still lack of specificity^36–39^.

Confounding factors influencing body temperature, heart and respiratory rates and white blood cell count included in SIRS comprehended beta-blockers, calcium-antagonists and other antiarrhythmic drugs or pace-maker DDD (heart rate), paracetamol, anti-inflammatory drugs and antimicrobials (body temperature); benzodiazepine, sedative and chronic oxygen administration (respiratory rate); immunosuppressive drugs and antimicrobials (white blood cell count). The presence of these factors could have a significant impact on clinical criteria positivity^40^.

Ideally, the best criteria should be as rapid as practically reliable for an early diagnosis and treatment of sepsis. In this prospective study, sepsis was diagnosed according to SIRS Criteria of 1991, Second Consensus Conference Criteria, modified Second Consensus Conference Criteria, Third Consensus Conference Criteria, in comparison with PCT and MR-proADM measurement. ROC curve analysis used to evaluate the diagnostic accuracy of the different criteria showed complete overlapping of the curves. On this basis, it should be convenient to prefer using bedside SIRS criteria or qSOFA in non-ICU setting rather than SOFA score requiring laboratory screening, Glasgow coma scale determination and knowledge of patients’ comorbidities or previous organ failures. Second Consensus Criteria of 2001 require the measurement of multiple clinical as well as bioumoral parameters needing long determination time. In this study, SIRS criteria and qSOFA allowed a diagnosis in 97% and 96% of patients, respectively, in case of suspicion of sepsis outside ICU. In the last years, plasma biomarkers have been proposed as tools for a rapid diagnosis and good indicator of prognosis. Among these, PCT and MR-proADM showed the best diagnostic and prognostic accuracy for the complementary nature of given information. PCT was optimal for etiological diagnosis and antimicrobial therapy management^12^, whereas MR-proADM was significantly correlated with organ failure and worse prognosis. In the present study, ROC analysis showed that besides clinical scores, PCT measurement represent the best diagnostic accuracy in sepsis, as previously described^12,21–24,41,42^ allowing early tailored antimicrobial therapy administration and daily follow-up. It should be reliable to combine bedside SIRS criteria or qSOFA with PCT laboratory determination for early identification of sepsis, followed by SOFA score calculation for severity and prognosis evaluation. In this study, about 35% of patients were negative for SIRS criteria or qSOFA, and SOFA score or for all, despite evidence of positive blood culture and documented microbiological isolate or clinical diagnosis of infection. In these patients, the use of MR-proADM was essential to provide early diagnosis and confirm the suspicion of sepsis.

These results suggest that in case of suspected sepsis, SIRS criteria or qSOFA should be bedside evaluated together with PCT measurement. These combinations reach a post-test probability of 99.9%. Besides PCT and MR-proADM, a marker of organ failure, even if comparable to SOFA score in sepsis severity prediction, showed the ability to anticipate SOFA and qSOFA score and the advantage to be more objective and fasten measured, as previously described outside ICU^24^. Exactly, in case of clinical suspicious of infection the presence of SIRS criteria ≥ 2, qSOFA ≥ 2, PCT ≥ 0.5 and MR-proADM ≥ 1.5 nmol/L identifies sepsis in 99.9% of cases. This approach, reliable in about 45 min, could allow an early diagnosis of sepsis within the first hour, even outside the intensive care contest to reduce the need for ICU transfer and mortality, as previously reported^24,43^.

Data from the prospective study highlighted comparable diagnostic accuracy between SIRS criteria from the First Consensus Conference of 1991, Criteria from the Second Consensus Conference of 2001 and from the Third Consensus Conference of 2016. Moreover, the use of the modified Second Consensus Conference Criteria of 2001, based on SIRS criteria plus SOFA score for sepsis diagnosis, did not improve diagnostic accuracy more than PCT and MR-proADM. In this study, SIRS criteria allowed a diagnosis in 97% of patient and, when combined with PCT measurement, identified 99.9% of septic patients. Moreover, MR-proADM values > 1 nmol/L showed the ability to identify septic patients when SIRS, SOFA and PCT were still negative. These results confirm those reported by other authors where MR-proADM anticipates by 24 h the organ failure development^44^.

Through SIRS criteria, qSOFA, PCT and MR-proADM determination, sepsis diagnosis can be achieved within the first hour from suspicion as recommended^35^ to improve outcome and decrease mortality. Furthermore, MR-proADM ≥ 1 nmol/L, even in case of negative PCT, qSOFA, SOFA, absence of Second Consensus Conference Criteria, identified septic patients with positive blood culture.

In conclusion, data from this study could suggest a diagnostic protocol for sepsis management outside ICU setting including, within 30 min from sepsis suspicion, bedside SIRS criteria or qSOFA score evaluation; within 1 h, PCT and MR-proADM measurement, microbiological culture collection, empiric sepsis therapy set up and SOFA score calculation. From these prompt actions, rapid diagnostic and prognostic evaluation of sepsis could be achieved also in case of negative SIRS, qSOFA or SOFA score with high post-test probability to reduce mortality and improve outcome.

### Informed consent

Written consent for publication was obtained from the patient before submission of this article.

## Abbreviations

APACHE: Acute Physiology and Chronic Health
AUCs: Areas under the curve
CBC: Complete blood counts
CRP: C-reactive protein
ICU: Intensive care unit
IL-6: Interleukin 6
MR-proADM: Mid-Regional pro-Adrenomedullin
PCR: Polymerase chain reaction
PCT: Procalcitonin
PPV: Positive predictive value
qSOFA: Quick
ROC: Receiver operating characteristic
SIRS: Systemic inflammatory response syndrome
SOFA: Sequential sepsis-related organ failure assessment
TRACE: Time-resolved amplified cryptate emission
WBC: White blood cell
